# Of mice and men: Dissecting the interaction between *Listeria monocytogenes* Internalin A and E-cadherin

**DOI:** 10.5936/csbj.201303022

**Published:** 2013-12-15

**Authors:** Samuel Genheden, Leif A Eriksson

**Affiliations:** a Division of Physical Chemistry, Department of Chemistry and Molecular Biology, University of Gothenburg, SE-41296, Gothenburg, Sweden; b Present address: School of Chemistry, University of Southampton, Highfield, SO17 1BJ, Southampton, UK

**Keywords:** Internalin A, E-cadherin, molecular dynamics, energy decomposition, umbrella sampling

## Abstract

We report a study of the interaction between internalin A (inlA) and human or murine E-cadherin (Ecad). inlA is used by *Listeria monocytogenes* to internalize itself into host cell, but the bacterium is unable to invade murine cells, which has been attributed to the difference in sequence between hEcad and mEcad. Using molecular dynamics simulations, MM/GBSA free energy calculations, hydrogen bond analysis, water characterization and umbrella sampling, we provide a complete atomistic picture of the binding between inlA and Ecad. We dissect key residues in the protein–protein interface and analyze the energetics using MM/GBSA. From this analysis it is clear that the binding of inlA–mEcad is weaker than inlA–hEcad, on par with the experimentally observed inability of inlA to bind to mEcad. However, extended MD simulations of 200 ns in length show no destabilization of the inlA–mEcad complex and the estimation of the potential of mean force (PMF) using umbrella sampling corroborates this conclusion. The binding strength computed from the PMFs show no significant difference between the two protein complexes. Hence, our study suggests that the inability of *L. monocytogenes* to invade murine cells cannot be explained by processes at the nanosecond to sub-microsecond time scale probed by the simulations performed here.

## Introduction

*Listeria monocytogenes* is a Gram-positive bacterium that causes listeriosis, a food-borne infection with a mortality rate up to 30%. Listeriosis causes meningo-encephalitis, gastroenteritis, and abortion in pregnant women. All of this is due to the ability of bacterium to cross the immune barriers of the host and to invade nonphagocytic cells. To invade host cells, *L. monocytogenes* uses two proteins of the internalin family and one of them, internalin A (inlA), is the focus of this study. InlA binds to the E-cadherin (Ecad) receptor on the host-cell surface, causing a cascade of signals that eventually leads to the internalization of the bacterium by the host cell [1–3].

**Figure 1:**
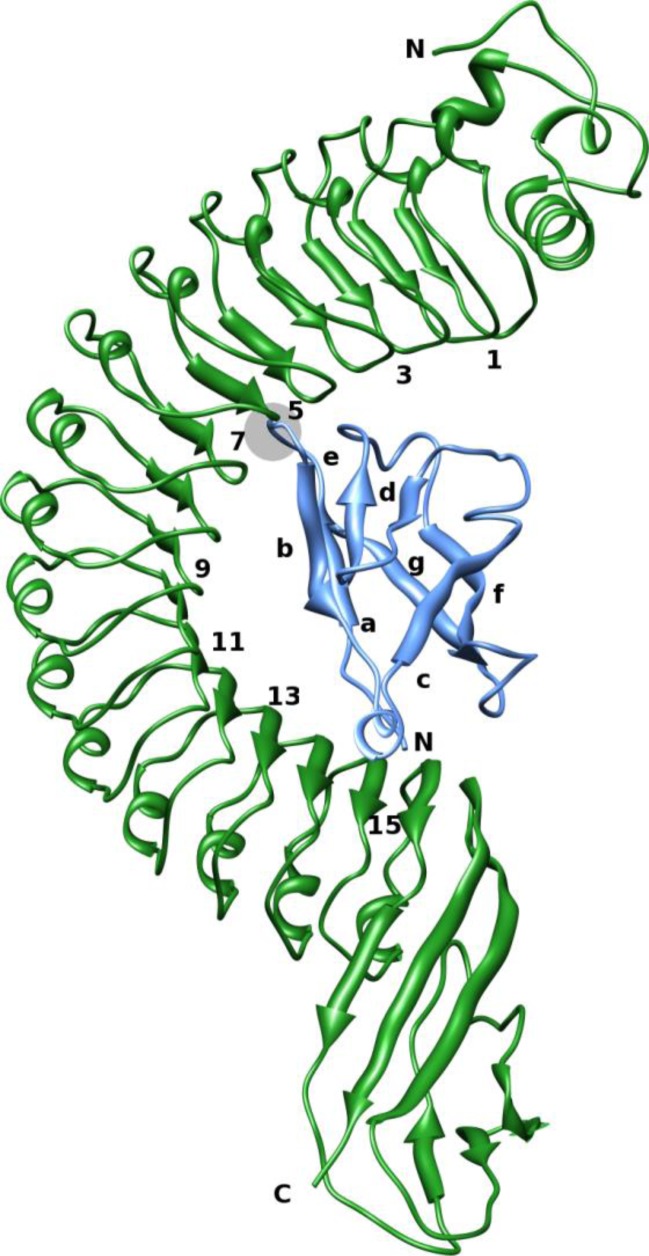
**Complex between inlA in green and hEcad in blue**. The numbering of every second inlA β-sheets(LRRs) is shown, as well as the numbering of the β-sheets in hEcad. N– and C–termini of the protein chains are marked with an N and C, respectively. The hEcad loop containing Pro16 and the tip of LRR 6 is encircled in grey.

The internalin family of proteins contains 25 proteins. All of these share a common architecture, including a signal peptide at the amino-terminus and several 22 amino acid leucine-rich repeats (LRR). The LRRs are followed downstream by several regions that are less conserved among the family members. InlA is an 800 amino-acid protein with 15 LRRs (see Figure 1) in the inter-repeat region that are fundamental for its biding to the Ecad, a motif for anchoring itself at the bacterial cell wall, and a sorting peptide at the carboxy-terminus [4, 5]. The crystal structure of inlA alone or in complex with the EC1 domain of Ecad has been solved [6].

**Figure 2:**
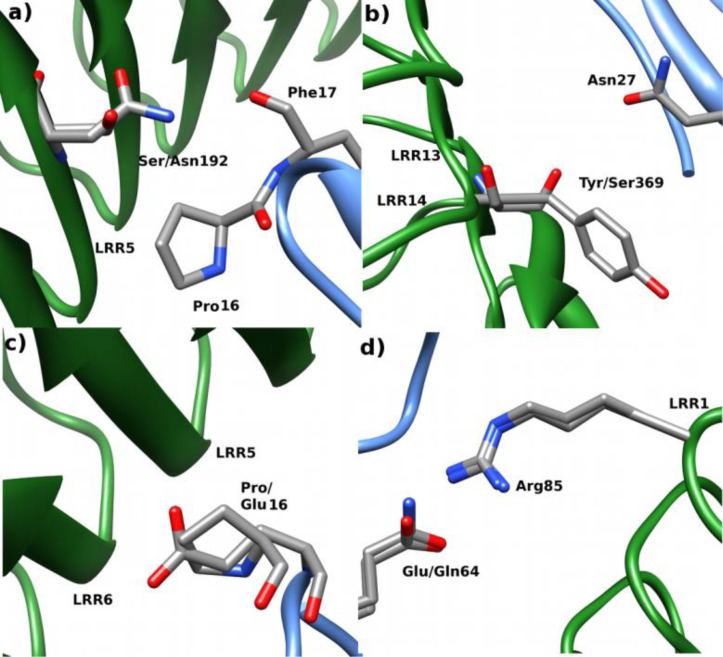
**Illustration of mutation in inlA, a) and b), and differences between hEcad and mEcad, c) and d)**. a) Illustrates the S192N mutation, which leads to an interaction between Asn192 and Phe17. b) Illustrates the Y369S mutation, which leads to an interaction with Asn27. c) Illustrates the important Pro/Glu16 difference between hEcad and mEcad. d) Illustrates the Glu/Gln64 difference.

Because of the emerging occurrence of *L. monocytogenes* in the industrialized world, it is important to understand how the bacterium invades the human cell. An important tool to study bacterial infections is to use animal models. Mouse is a popular model because it is eukaryotic but a much simpler species than human [7, 8]. However, *L. monocytogenes* does not invade mouse cells at the same rate as humans, all because the binding between inlA and murine Ecad is too weak for the bacterium to adhere to the cell surface [3, 6]. This observation has spurred research on the interface between inlA and Ecad to determine key interactions that are present in the human but not the murine case. One key residue on human Ecad (hEcad) that has been identified is Pro16 that is mutated to a Glu in murine Ecad (mEcad). In hEcad, the apolar proline binds in a neutral and hydrophobic cavity on inlA at LRR loop 6 (see Figure 1) [6]. Therefore, it has been hypothesized that the larger and charged Glu cannot fit in the cavity in addition to lacking any clear interaction partner, resulting in impaired inlA–mEcad interaction. Other key interactions have been hypothesized and tested with mutant proteins [9, 10]. The Y369S and S192N mutations on inlA have been shown to improve the affinity for hEcad, especially if they are introduced simultaneously, by improving the interfacial interactions. Furthermore, the Q64E mutation on mEcad has shown to improve the interaction with inlA, but only if the E16P mutation is also introduced. These two mutations correspond to the conversion of the mEcad sequence to hEcad at the specific sites. All mutations are illustrated in Figure 2.

In this contribution, we will dissect the interaction between inlA and Ecad using computational tools, including investigation of both wild type and various mutant systems. By using a combination of molecular dynamics, free energy calculations, hydrogen-bond analysis, and water site characterization, we will present a detailed description of the interface at an atomistic level. Such techniques have been readily used to study several protein–protein interfaces [11–14]. We not only reveal the energetics of the protein–protein interaction, but we also show that energetic differences alone between the inlA–hEcad and inlA–mEcad complexes are not sufficient to describe the inability of inlA to invade murine cells.

## Methods

### System preparation

The complex between *L. Monocytogenes* Internalin A (inlA) and either human or murine E-cadherin (hEcad and mEcad, respectively) was simulated. The inlA–hEcad complex is shown in Figure 1, together with numbering of the β-sheets (LRRs). Both wild-type (WT) and various mutants were simulated, based on several available crystal structures as outlined in Table 1. If a complex did not exist in the PDB database, it was created from an available crystal structure by modifying the side-chain of the amino acid(s) *in silico*. All protein residues were described with the Amber99SB-ILDN force field [15]. The side-chains were set to normal protonation states at pH 7, i.e., all Arg and Lys residues were positively charged, and all Glu and Asp residues were negatively charged. The protonation state of the histidine residues were decided by considering hydrogen-bond networks. Hence, His392 in InlA was protonated on the NE2 atoms, and His79 in Ecad was protonated on the ND1 atom. The complexes were solvated in a rectangular box of pre-equilibrated TIP3P water molecules [16], extending at least 9 Å from the solute. In total, ∼95,000 atoms were simulated in each system. A few simulations as described in the text used a larger box, extending at least 15 Å from the solute. In those cases, a total of ∼135,000 atoms were simulated.

**Table 1:** Complexes in this study. The mutants to inlA and Ecad are indicated, as well as the crystal structures on which the simulations were based.

inlA mutation	Ecad mutation	Crystal structure	Equilibration length (ns)^b^
**inlA-hEcad complexes**			
-	-	1O6S^a^	1
Y369S	-	1O6S	4
S192N	-	2OMY^a^	1
Y369S / S192N	-	2OMV^a^	1
-	P16E	1O6S	4
-	E64Q	1O6S	4
-	P16E / E64Q	1O6S	4
Y369S / S192N	P16E / E64Q	2OMV	4

**inlA-mEcad complexes**			
-	-	2OMW	4
Y369S / S192N	-	2OMW^a^	1
Y369S / S192N	E16P	2OMW	4
Y369S / S192N	Q64E	2OMW	4
Y369S / S192N	E16P / Q64E	2OMW	4
-	E16P / Q64E	2OMW	4

a This complex was in the crystal structure

b Equilibration length of the MM/GBSA simulations. This was followed by a 1 ns production run.

### Simulations

All simulations were performed using Gromacs v4.5.5 [17]. All bonds involving hydrogen atoms were constrained using the LINCS algorithm [18], and the time step of the integration of motions was 2 fs. The non-bonded cut-off was 9 Å, and the non-bonded pair-list was updated every 50 fs. Electrostatic interactions were treated using particle-mesh Ewald summation [19], and long-range van der Waal interactions were corrected using a continuum approach [20]. The temperature was kept at 300 K using a velocity re-scaling algorithm with a stochastic term [21] and a coupling constant of 1 ps. The pressure was kept constant at 1 atm using a weak-coupling [22], isotropic algorithm with a coupling constant of 1 ps.

Ten independent simulations were generated for each complex by solvating the complex in different boxes of pre-equilibrated solvent and by assigning different initial velocities [23]. Each of the ten independent simulations were first minimized using 500 steps of minimization with harmonic restraints of 200 kJ/mol on protein non-hydrogen atoms, followed by a 100 ps simulation in the NPT ensemble using the same restraints. Thereafter, the systems were equilibrated in the same ensemble but without restraints for 1000 ps if the complex did exists as a crystal structure, or 4000 ps if the complex was created by modifying a crystal structure. The equilibration was followed by a 1000 ps production run in the NPT ensemble, where snapshots were extracted every 5 ps. Hence, from each simulation, 200 snapshots were extracted for analysis.

### Free energy calculations

The free energy of binding between inlA and Ecad was estimated using MM/GBSA (molecular mechanics with generalized Born – surface area) [24], with the mmgbsa.py script in AmberTools12 [25]. The free energy is expressed as the difference in free energy between the complex and the two binding partners, i.e., ▵*G* = ▵*G*(inlA–Ecad) – ▵*G*(inlA) – ▵*G*(Ecad), and each of these free energies are calculated as [24]

$$G=<E_{\mathrm{int}}+E_{\text{ele}}+E_{\text{vdw}}+\Delta G_{\text{pol}}+\Delta G_{\text{np}}-TS>$$

where the first three terms are the molecular mechanics internal, electrostatic and van der Waals energy, respectively; ▵*G*_pol_ and ▵*G*_np_ are the polar and non-polar solvation free energy, and *T* and *S* is the absolute temperature and an entropy estimate. The brackets indicate an average over an ensemble of snapshots from the MD simulations. Here, we make a common approximation and evaluate the free energy of free inlA and Ecad from the complex simulation, because of the improved precision [26]. Thereby, the *E*_int_ term cancels. Furthermore, because accurate calculation of the entropy term is extremely costly for such a large protein–protein complex, and because we cannot easily decompose the entropy, it will be ignored herein. For relative free energies of similar systems, this has been shown to be a good approximation [27].

The energy terms were evaluated using the same force field as in the simulations, but without any non-bonded cut-off. The ▵*G*_pol_ term was evaluated using the generalized Born method of Onufriev, Bashford and Case, model I [28]. The ▵*G*_np_ term was evaluated through a linear relation to the solvent accessible surface area (SASA), i.e., γSASA, with γ = 0.03 kJ/mol [29]. The free energy for each system was evaluated using 200 snapshots from 10 independent simulations, i.e., 2,000 snapshots in total. The reported uncertainties are the standard deviation of the mean over the 10 independent simulations.

MM/GBSA was also used to perform alanine-scanning mutagenesis (ASM) [11]. In ASM, the free energy of mutating one amino acid to an alanine is computed. Here, we used the common single-trajectory approach [25], i.e., the mutated residue was estimated using the ensemble of snapshots generated with the original residue. We also tested a variant of ASM, which we will denote scaled ASM (sASM) [27, 30]. In this approach the internal dielectric of the protein used in calculating electrostatic and polar solvation terms is scaled to correct for the fact that we use a single-trajectory approach and thereby ignore the protein reorganization energy. For apolar amino acids, the scaling factor is two, for polar and uncharged amino acids three, and for charged amino acids four.

### Hydrogen-bond analysis

Hydrogen bond analysis was performed on the same 2,000 snapshots per system that were used for the MM/GBSA analysis. We analyzed hydrogen bonds between residues in inlA and residues in Ecad, as well as between interfacial residues and water molecules. Interfacial residues were determined to be residues in inlA that had an atom at most 4 Å from a residue in Ecad, and vice versa. The crystal structure of inlA–hEcad was used to calculate the distances. The threshold for finding hydrogen bonds was a length of 3.5 Å between the heavy atoms and an angle cut-off of 135°.

### Water analysis

Conserved water sites in the interface were found using a clustering algorithm [31]. Each MD snapshot was superposed onto the crystal structure by fitting the backbone heavy atoms of each residue within 8 Å of the interfacial residues. (Interfacial residues defined as in the hydrogen bond analysis.) Then, oxygen atoms of water molecules within 3 Å of the interfacial residues were saved for clustering. When all snapshots had been processed, the stored water molecules were clustered. The water molecule with the largest number of water molecules within 1 Å was defined to be the center of a conserved water site, and this water molecule and all water molecules within 1 Å were removed from further analysis. This procedure was repeated until the number of water molecules found at a site was lower than what is expected from a bulk water simulation.

The interaction energy between each water molecule in the cluster and the rest of the system was monitored. An entropy estimate for each site was calculated from inhomogeneous solvation theory [32, 33] by considering the internal translational and rotational entropy. Hence, water–water correlation was ignored. The translational entropy was calculated by assuming a uniform distribution and the rotational entropy was calculated by considering the rotation of Euler angles using an approach outlined recently [34].

### Umbrella Sampling

**Figure 3:**
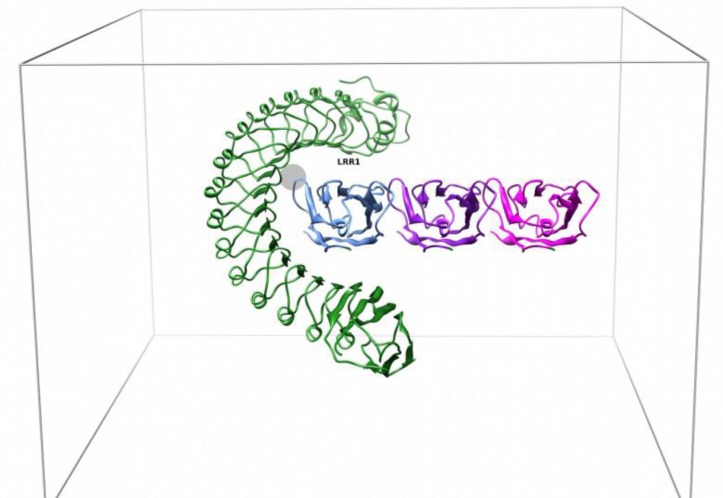
**Illustration of the direction of displacement in the umbrella calculations**. inlA is shown in green at one edge of the simulation box. The position of hEcad as observed in the crystal structure is then shown in blue, and at displacements of 24 Å and 48 Å in purple and pink, respectively. The simulation box is sketched for reference. The hEcad loop containing Pro16 and the tip of LRR 6 is encircled in grey.

A potential of mean force (PMF) between inlA and hEcad or mEcad was calculated using umbrella sampling [35]. The complex was placed in a 95x135x130 Å box such that it was roughly 10 Å from the edge of the box in all directions. Next, either hEcad or mEcad was displaced from inlA at specific center-of-mass distances in the y-direction (illustrated in Figure 3). Displacements of 0, 1, 2, 3 Å, and then in 2 Å intervals, for displacements up to 48 Å were used. At each displacement, the complex was solvated with TIP3P water molecules. In total ∼165,000 atoms were simulated. The complex was subsequently simulated at each value of displacement and the center-of-mass distance in the y-direction was enforced with a harmonic potential with a force constant of 1000 kJ/mol (this magnitude gives a good overlap of the distance distributions between individual simulations). The simulations were performed as described above for the unconstrained MD simulations. The systems were equilibrated for 2 ns before a 6 ns production run. The PMFs were then estimated by the weighted histogram analysis method [36] implementation in Gromacs [37].

## Results

We have simulated the complex between iternalin A (inlA) and either human or murine E-cadherin (hEcad or mEcad) using molecular dynamics. Both wild-type (WT) systems and a range of mutants have been simulated. In what follows, we will present the results of the various analyses performed on the generated trajectories.

### MM/GBSA free energies

The binding free energies between the inlA and Ecad in the various complexes were estimated by MM/GBSA and are given in Table 2. It should be noted that conformational entropy was neglected, as mentioned above, an approximation that has often been used when studying protein–protein complexes [12, 13, 30]. An RMSD analysis (see Table S1) indicated that the simulations were sufficiently stable. The affinity of WT inlA–hEcad is –207 kJ/mol compared to the affinity of inlA–mEcad that of only –152 kJ/mol, consistent with experiments. The uncertainty is rather high, indicating that the total free energy is not fully converged. However, as we will see, this has minor importance when considering individual residues. By decomposition, we can obtain an estimate of how each species contribute to the total free energy. It is clear from Table 2, that in general a majority of the binding free energy comes from Ecad, although the ratio is close to 50%.

**Table 2:** MM/GBSA free energy estimates of inlA–Ecad complexes in kJ/mol.

inlA mutation	Ecad mutation	▵*G* a	▵*G* (inlA)^b^	▵*G* (Ecad) c
**inlA–hEcad complexs**				
-	-	-207.4 ± 7.4	-93.1	-114.4
Y369S	-	-180.5 ± 13.0	-73.9	-106.7
S192N	-	-207.8 ± 6.6	-94.7	-113.1
Y369S / S192N	-	-201 ± 5.9	-88.5	-112.5
-	P16E	-173 ± 13.7	-103.8	-69.2
-	E64Q	-174.7 ± 9.4	-63	-106.4
-	P16E / E64Q	-166.2 ± 9.1	-87.9	-78.3
Y369S / S192N	P16E / E64Q	-151.5 ± 6.4	-80.8	-70.7

**inlA–mEcad complexs**				
-	-	-152.1 ± 5.9	-72.1	-80
Y369S / S192N	-	-169.4 ± 7.5	-79.8	-89.6
Y369S / S192N	E16P	-164.7 ± 7.9	-59.1	-105.6
Y369S / S192N	Q64E	-177.4 ± 6.7	-94.3	-83.1
Y369S / S192N	E16P / Q64E	-176.2 ± 10.2	-75.2	-101
-	E16P / Q64E	-175.6 ± 7.8	-75.2	-100.5

a ▵*G* = ▵*G*(inlA–Ecad) – ▵*G*(inlA) – ▵*G*(Ecad) according to the MM/GBSA formulation.

b Component of ▵*G* arising from inlA.

c Component of ▵*G* arising from Ecad.

We then simulated a number of different mutants to probe key interactions in the interface that have been explored experimentally. The inlA mutations Y369S and S192N have been shown experimentally to improve the binding between inlA and Ecad. However, the simulations with these mutations or the double mutant predict a reduced affinity by up to 27 kJ/mol for the inlA–hEcad complex. Because of the large uncertainty, the differences are not statistically significant. For the S192N / Y369S double mutant simulation of the inlA–mEcad complex, the affinity is increased by 17 kJ/mol.

Two residues on mEcad have been probed experimentally, namely Glu16 and Gln64. Mutating Glu16 to Pro16, gives a 13 kJ/mol more negative free energy estimate, in accordance with experiments (but not statistically significant). Likewise, mutating Gln64 to Glu64, give a 25 kJ/mol more negative binding affinity, and the double mutant E16P/Q64E also gives a significant 24 kJ/mol more negative binding affinity, irrespective of whether the inlA S192N / Y369S double mutant is introduced or not. It is interesting to note that the largest change to the binding affinity when introducing the Q64E mutation on mEcad comes from inlA, not from mEcad as one would suppose.

To check the importance of these residues, we introduced reverse mutations on the inlA–hEcad complex (i.e. modifying hEcad towards mEcad). Both the P16E and E64Q mutants give statistically significant reduced binding affinities, by 34 and 33 kJ/mol respectively. The double mutant gives an even more reduced binding affinity (41 kJ/mol), and if the S192N / Y369S double mutant is also introduced on inlA, this reduces even further. Introducing the E64Q mutation gives rise to a large change in the contribution from inlA, but only a moderate change in the contribution from Ecad, whereas for the P16E mutant the opposite is found. This complements perfectly the opposing trends seen for the E16P and Q64E mutants in mEcad.

### Free energy decomposition and alanine-scanning mutagenesis

**Figure 4:**
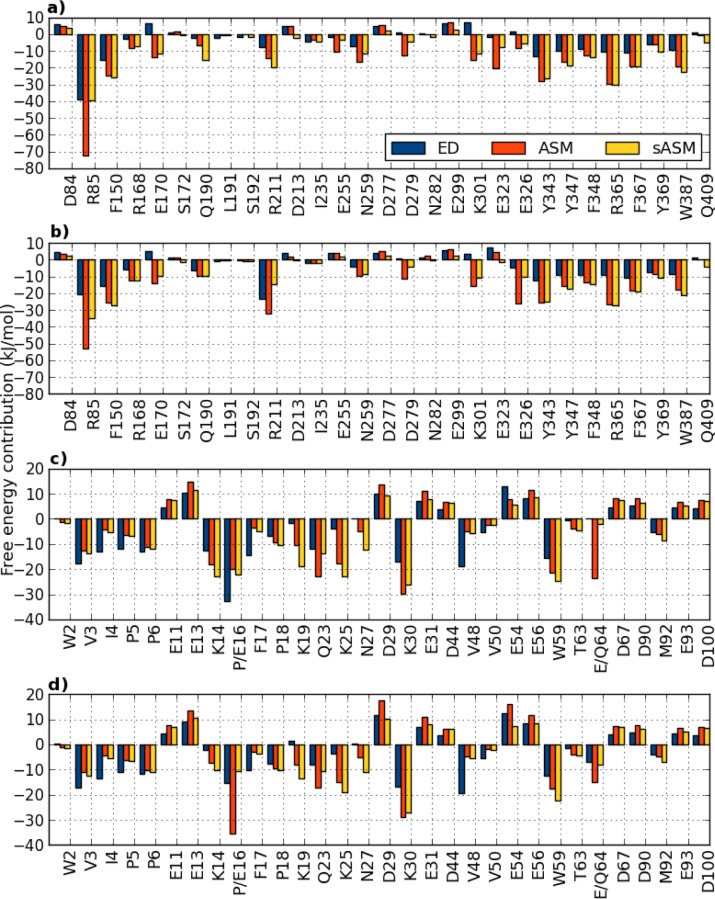
**Free energy contributions of residues on inlA and Ecad in kJ/mol**. a) inlA residues in the inlA–hEcad complex, b) inlA residues in the inlA–mEcad complex, c) Ecad residues in the inlA–hEcad complex, d) Ecad residues in the inlA–mEcad complex. Residues were selected based on a number of criteria as outlined in the text. Free energy contributions are determined by energy decomposition (ED), alanine scanning mutagenesis (ASM), and scaled ASM (sASM).

The total binding free energy was decomposed on a residue-wise basis to determine which residues that are most important for binding. The free energy contributions from all residues are plotted in Figure 4 (and shown in Table S2). For the residues on inlA, the major contribution comes from a few residues throughout the sequence, and there are a few distinct differences between the inlA–hEcad and inlA–mEcad complexes. Interestingly, the charged residues Arg85, Arg211, Glu255, Glu323, and Glu326 all show a difference in interaction with hEcad vs. mEcad larger than 5 kJ/mol, when comparing the two complexes. Instead looking at the residues on Ecad, it is clear that many of them display large contributions (see Figure 4 and Table S3). However, when summing up the difference between inlA–hEcad and inlA–mEcad, most of the residue contributions cancel. Only residues Lys14, Gly15, Pro/Glu16, and Glu/Gln64 show a difference larger than 5 kJ/mol.

An alternative to this energy decomposition (ED) is alanine-scanning mutagenesis (ASM), in which the effect on the free energy of mutating a particular residue to alanine is estimated. ASM is more expensive than ED, and it is therefore not feasible to perform ASM on all residues in the complex. To determine which residues on which to perform ASM, we used a number of criteria. First, the residue should have an ED contribution of more than 4 kJ/mol in either the inlA–hEcad or inlA–mEcad complex. Second, the residue should be an interfacial residue, i.e. it should be within 4 Å of a residue on the other protein. Third, the residue is identified as a hydrogen-bonding partner (see below). Fourth, and last, the residue has been discussed in the literature to be important for the binding. If at least one of the criteria is fulfilled, ASM and scaled ASM (sASM) were computed, with the exception of Glycine residues as well as N– and C–terminal residues (due to limitation in mmpbsa.py). The residues on inlA and Ecad identified in this way are included in displayed in Figure 4 and listed in Tables S2 and S3.

For inlA, 29 residues were detected using the above criteria. Of these, 14 are charged residues, nine are uncharged but polar, and six are apolar. It is common to introduce a threshold to determine the most important residues, usually called hot or warm spots [13]. There are different definitions of this; here we use a threshold of 8 kJ/mol to determine hot spots, i.e., all residues that have an absolute ED contribution or an ASM or sASM absolute free energy of greater than 8 kJ/mol are considered to be important. Unfortunately, ED, ASM, and sASM do not always agree. This is not surprising, as the method use different levels of approximations. For the inlA–hEcad complex, ED distinguishes eight hot spots, ASM 17, and sASM 14, and only on eight residues do the methods completely agree. However, if we use the argument that it is sufficient that two methods agree, we can identify twelve hot spots on inlA for the inlA–hEcad complex and 16 for the inlA–mEcad complex. For the inlA–hEcad complex, the hot spots are Arg85, Phe150, Glu170, Arg211, Asn259, Lys301, Tyr343, Tyr347, Phe348, Arg365, Phe367 and Trp387. Most of these residues are either charged or polar. For the inlA–mEcad complex, the hot spots are Arg85, Phe150, Arg168, Glu170, Gln190, Arg211, Asn259, Lys301, Glu326, Tyr343, Tyr347, Phe348, Arg365, Phe367, Tyr369 and Trp387. Hence, Arg168, Gln190, Glu326, and Tyr369 were identified as hot spots on inlA–mEcad but not on inlA–hEcad. In total, the hot spots contribute –118 and –139 kJ/mol, to the inlA–hEcad and inlA–mEcad affinities, respectively.

For Ecad we identified 34 residues using the criteria above, and 31 on which to perform ASM. For hEcad, we found 16 charged residues, four uncharged but polar, and 14 apolar residues. For mEcad, there were 16 charged, three polar, and 15 apolar residues. Of these, we identified 13 and 15 hot spots on the inlA–hEcad and inlA–mEcad complexes respectively. For inlA–hEcad, the hot spots are Val3, Pro6, Glu13, Lys14, Pro16, Pro18, Lys19, Gln23, Lys25, Asp29, Lys30, Glu56, and Trp59. For the inlA–mEcad, the hot spots are Val3, Pro6, Glu13, Glu16, Pro18, Lys19, Gln23, Lys25, Asp29, Lys30, Glu31, Glu56, Trp59, and Gln64. Hence, Lys14 is a hot spot in the inlA–hEcad complex but not in inlA–mEcad, and Glu31 and Gln64 are hot spots in the inlA–mEcad complex, but not in inlA–hEcad; both Lys14 and Glu31 are however close to being hot spots in both complexes. The hot spots contribute –104 and –50 kJ/mol, to the inlA–hEcad and inlA–mEcad affinities, respectively.

**Figure 5:**
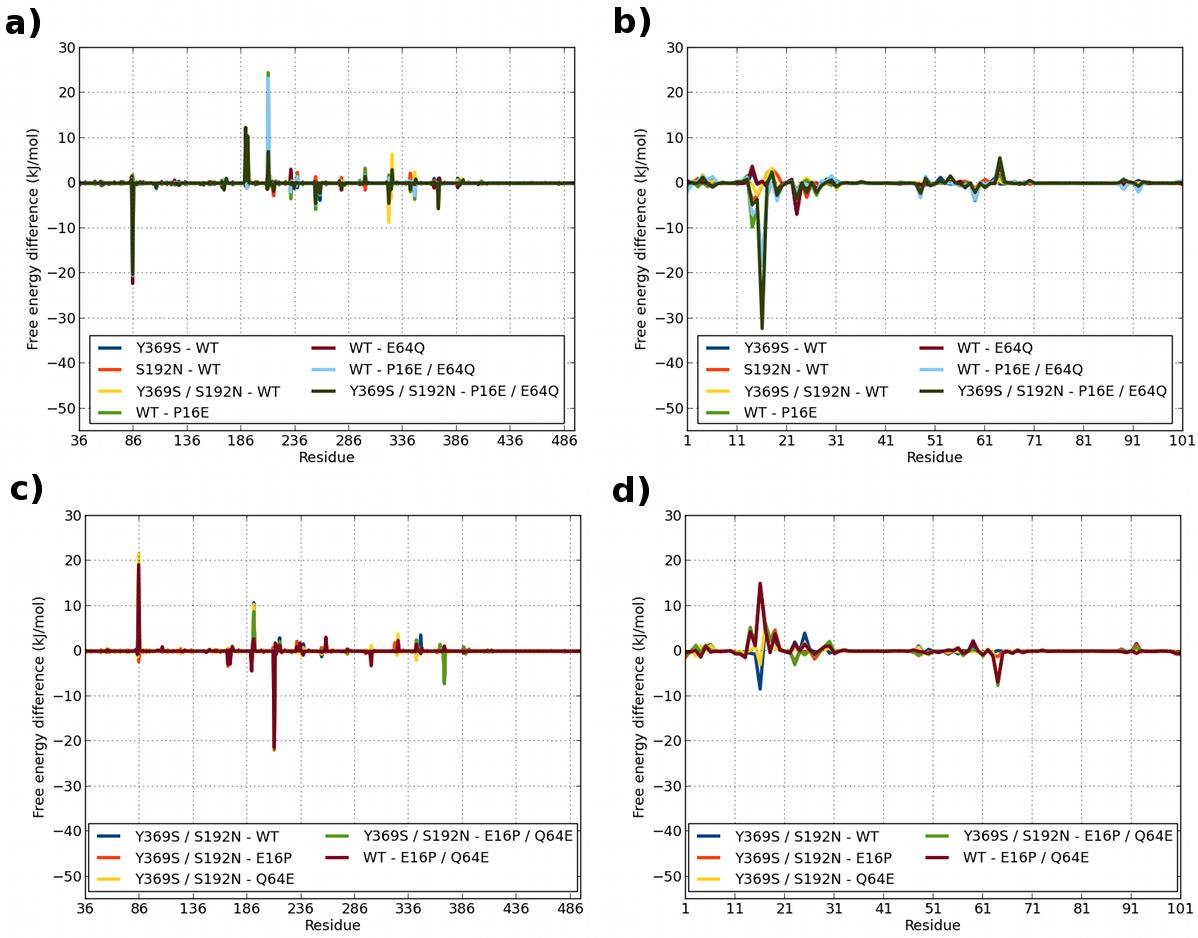
**Per-residue free energy difference between wild-type inlA–Ecad complex and various mutants**. a) Difference relative to inlA–hEcad complex for residues on inlA, b) Difference relative to inlA–hEcad complex for residues on Ecad, c) Difference relative to inlA–mEcad complex for residues on inlA, d) Difference relative to inlA–mEcad complex for residues on Ecad

In Figure 5, we have plotted the residue-by-residue difference (corresponding to ED in Tables S1 and S2) for the mutant simulations, compared to the WT simulation. A negative value implies that the residue has a more negative binding free energy in WT than in the mutant. In accordance with the small effect of the mutants Y369S and S192N on the binding energies, very few residues show a large difference for these two mutants. In addition, introducing the E16P and Q64E mutations on mEcad, gives surprisingly few changes throughout either inlA or mEcad. Only three residues on inlA and only two residues on mEcad show a difference larger than 5 kJ/mol. Introducing the double mutant E16P / Q64E, gives a few more residues with a difference larger than 5 kJ/mol. Likewise, if we introduce the reverse mutation on the inlA–hEcad complex, we only see a few changes for the P16E and E64Q mutations. The changes are highly localized around the respective mutations.

### Hydrogen-bond analysis

The inter-protein hydrogen bonds between inlA and Ecad were monitored throughout the simulations. The hydrogen bonds with an average occupancy of more than 10% are listed in Table 3. Starting with interactions between a backbone donor/acceptor and a side chain acceptor/donor, we identified five hydrogen bonds. The backbone oxygen atom of Ile4 on Ecad forms a very clear hydrogen bond with the side chain of Arg365 on inlA, in both the inlA–hEcad and the inlA–mEcad complexes. The same interaction was observed between Val48 on Ecad and the Arg85 side-chain on InlA, although the occupancy is much lower. Furthermore, the backbone nitrogen atom of Phe17 in Ecad donates a hydrogen atom to the side chain of Glu170 in inlA, in both complexes. However, the hydrogen bond between the backbone oxygen atom of Gly15 in Ecad to the side-chain of Arg211 in inlA is only formed in the inlA–hEcad simulation.

**Table 3:** Hydrogen bonds between inlA and Ecad.

Donor			Acceptor			inlA–hEcad complex		inlA–mEcad complex	
Protein	Residue	Atom	Protein	Residue	Atom	Occurance^a^	Occupancy^b^	Occurancea	Occupancy^b^
**Backbone – side chain interactions**									
Ecad	Ile4	O	InlA	Arg365	NH1	10	83.4 ± 4.2	10	79.3 ± 8.9
Ecad	Gly15	O	InlA	Arg211	NE	10	70.2 ± 5.5		
Ecad	Val48	O	InlA	Arg85	NE	10	93.6 ± 0.9	10	87.5 ± 2.2
					NH2	10	40.9 ± 4.6	10	68.2 ± 3.1
inlA	Gly170	OE2	Ecad	Phe17	N	10	86.6 ± 0.7	9	79.1 ± 1.3

**Side chain – side chain interactions**									
Ecad	Glu16	OE1/OE2	InlaA	Arg211	NE			10	44.7 ± 7.2
		OE2			NE2			5	27 ± 8.6
		OE1/OE2			NH2			8	46.2 ± 13.5
Ecad	Gln23	OE1	InlA	Asn259	ND2	9	32 ± 2.9	9	15.2 ± 4.5
				Lys301	NZ	6	46.1 ± 11.6	8	30.4 ± 8.2
Ecad	Glu54	OE1/OE2	InlA	Ser216	OG	8	27.8 ± 11.8	4	4.1 ± 2.0
Ecad	Glu/Gln64	OE1/OE2		Arg85	NH1/NH2	10	39 ± 9.0	10	50.6 ± 5.4
inlA	Glu255	OE1/OE2	Ecad	Lys19	NZ	6	12.8 ± 3.7		
inlA	Asn259	OD1	Ecad	Trp59	NE1	10	26 ± 3.2	6	7.4 ± 1.5
inlA	Asn282	OD1	Ecad	Gln23	NE2	5	16.8 ± 9.5		
inlA	Glu323	OE1/OE2	Ecad	Lys25	NZ	9	12.7 ± 3.7		
inla	Glu326	OE1/OE2	Ecad	Lys25	NZ			6	21.6 ± 5.2
			Ecad	Lys30	NZ	9	15.8 ± 4.2	8	20.1 ± 5.6

a The number of simulations in which the hydrogen bond occurred

b The average number of snapshots the hydrogen bond was formed

Looking at side-chain-to-side-chain interactions, we find nine hydrogen bonds in the inlA–hEcad complex, and ten in the inlA–mEcad complex. Certain hydrogen bonds are formed in inlA–hEcad only, namely between Glu255 on inlA and Lys19 on Ecad, between Asn282 on inlA and Gln23 on Ecad, and between Glu323 on inlA and Lys25 on Ecad. Likewise, hydrogen bonds between Glu16 on Ecad and various nitrogen atoms of Arg211 on inlA are only formed in the inlA-mEcad complex. These are naturally not possible in inlA–hEcad due to the presence of Pro16 in that case. The hydrogen bonds between Glu54 on Ecad and Ser216 on inlA, as well as between Asn259 on inlA and Trp59 on Ecad are formed in both complexes, but in inlA–mEcad, the hydrogen bonds are formed with a very low average occupancy. Furthermore, Gln23 on Ecad forms a hydrogen bond with Asn259 and Lys301 on inlA in both complexes. The same is true for Glu/Gln64 on Ecad and Arg85 on inlA. Lastly, Glu326 on inlA makes a hydrogen bond with Lys25 on Ecad in the inlA–mEcad complex, and with Lys30 in both the inlA–hEcad and inlA–mEcad complexes.

### Conserved water sites

We identified conserved water sites by clustering water molecules in the interface between inlA and Ecad. In Table 4 we list the water sites with occupancy of at least 25%, i.e., that occurred in at least 400 of the 2000 snapshots saved for the 10 independent simulations. We identified 26 such sites in the inlA–hEcad complex, and 18 sites in the inlA–mEcad complex. The average interaction energy of the water sites in the inlA–hEcad complex ranges from –33 to –95 kJ/mol, with an average of –63 kJ/mol. For the inlA–mEcad complex, the average interaction energy of the water sites shows a much larger range from +1 to –90 kJ/mol, with an average of –52 kJ/mol. The total internal entropy of the sites is positive for all sites and is dominated by the rotational entropy (not shown). It ranges from 12 to 27 kJ/mol for the inlA–hEcad complex and from 10 to 22 kJ/mol for the inlA–mEcad complex.

**Table 4:** Conserved water sites in the interface^a^.

Occupancy	Cluster	Interaction energy^b^	Entropy^c^	Close residues^d^				
**inlA–hEcad complex**								
1498	2	-90.8 ± 0.7	27.3	Ser192a	Pro16b			
1223	3	-79.9 ± 1.0	23.5	Arg85a	Asn107a	Asn128a	Thr63b	
1193	1	-77.2 ± 0.9	23.3	Lys301a	Glu323a	Lys25b		
1183	2	-56.2 ± 1.1	21.4	Arg211a	Pro16b	Lys19b		
1126	1	-67.1 ± 1.1	23.9	Lys301a	Val22b	Gln23b		
1107	1	-94.6 ± 1.0	22.2	Tyr347a	Lys25b	Asn27b		
960	2	-64.6 ± 0.8	13.9	Arg168a	Gln190a	Gly15b	Phe17b	
937	2	-81 ± 0.6	17.4	Phe150a	Glu170a	Ser172a	Ser192a	Phe17b
932		-32.8 ± 0.8	17.9	Arg85a	Pro46b	Pro47b	Val48b	
904	1	-74.4 ± 1.0	21.5	Tyr343a	Val3b			
881	1	-61.9 ± 1.3	20	Glu323a	Pro5b			
835	2	-51.9 ± 0.5	14.4	Arg211a	Ser233a	Pro16b		
801	2	-66.7 ± 1.0	13.4	Asp213a	Pro16b			
777	2	-46.3 ± 0.9	20.1	Thr148a	Arg168a	Glu170a	Phe17b	
728	2	-53.6 ± 1.4	17.6	Ile235a	Lys19b	Asn20b		
711	2	-68.2 ± 1.2	17.9	Gln190a	Lys14b			
701	1	-55.5 ± 1.6	18.7	Asn259a	Ala281a	Lys301a	Gln23b	Lys25b
693	3	-60.5 ± 1.3	18	Arg85a	Asn104a	Ser106a	Glu64b	
677	1	-55.1 ± 0.9	17.1	Glu323a	Lys25b			
643	2	-39.5 ± 2.2	18	Ile235a	Glu255a	Lys19b	Asn20b	
514	2	-45.8 ± 1.3	16.2	Gln190a	Lys14b	Gly15b		
501		-58.9 ± 2.9	14.2	Arg365a	Pro6b			
486	1	-51.7 ± 1.7	14.6	Asn259a	Asn282a	Gln23b		
471	2	-44.7 ± 5.2	13.1	Ile235a	Glu255a	Lys19b		
468	1	-94.4 ± 1.6	11.6	Glu323a	Asn325a	Tyr347a	Lys25b	
423	1	-51.6 ± 1.6	13.3	Phe348a	Asn27b			

**inlA–mEcad complex**								
1251	2	-83.1 ± 0.9	16.5	Glu170a	Ser192a	Phe17b	Pro18b	
1029	1	-67.5 ± 1.3	21.9	Tyr347a	Val3b			
851	2	-66.1 ± 2.6	18.2	Ser172a	Ser192a	Pro18b		
845	1	-65.8 ± 1.2	17.1	Lys301a	Glu323a	Gln23b		
833	3	-58.2 ± 0.9	18	Arg85a	Asn107a	Asn128a	Thr63b	Gln64b
775	1	-80.9 ± 1.2	17.3	Glu323a	Tyr343a	Val3b	Pro5b	
740	1	-6.1 ± 4.7	20.2	Asn259a	Lys301a	Gln23b		
721	2	-38.4 ± 2.7	18.1	Phe150a	Ser172a	Ser173a	Pro18b	
684	2	-30 ± 1.4	17.5	Arg211a	Asp213a	Glu16b		
615	2	-24.8 ± 1.3	16.1	Leu191a	Arg211a	Glu16b		
588	1	-64.1 ± 1.5	14.6	Lys301a	Glu323a	Pro5b		
535	1	-46.9 ± 3.6	14.6	Lys301a	Gln23b	Trp59b		
532	1	-45.3 ± 2.3	10.8	Asn259a	Gln23b			
531	1	-89.9 ± 3.0	16.5	Glu326a	Tyr347a	Lys25b	Asn27b	
479	2	0.4 ± 4.4	11.7	Glu170a	Leu191a	Ser192a	Glu16b	
447	2	-44.3 ± 1.3	10.8	Arg211a	Ile235a	Glu16b		
444		-66 ± 1.2	13	Arg365a	Pro6b			
442	2	-54.3 ± 1.2	10	Thr148a	Glu170a	Phe17b		

a Showing sites with an occupancy of more than 400, i.e., it occurred in at least 25% of the snapshots.

b The interaction energies are in kJ/mol.

c The entropies are the sum of the internal translational and rotational entropies and given in kJ/mol

d A residue ending with an *a* is a residue on inlA, a residue ending with a *b* is a residue on Ecad. A threshold of 3 Å was used to define proximity.

**Figure 6:**
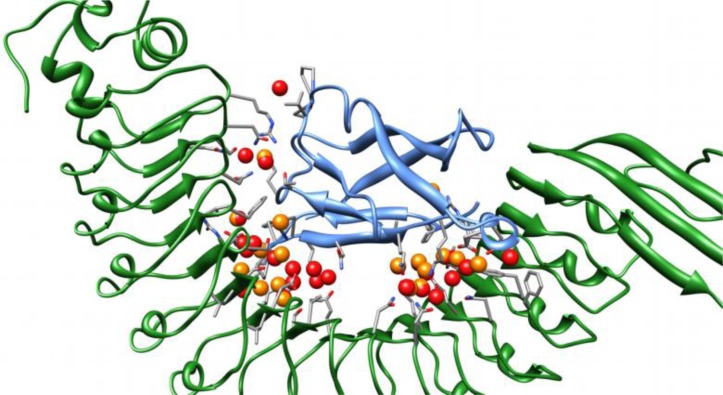
**Conserved water sites in the interface**. Showing the location of the sites listed in Table VI. The green protein is inlA and the blue protein is Ecad. Sites are shown as red and orange spheres, the red were found for the inlA–hEcad complex and the orange for the inlA–mEcad complex. Residues with 3 Å of the sites are shown as well.

The water sites are displayed in Figure 6, and clearly show that a majority of these are located in two large and one smaller clusters. One of the clusters is close to residues Asn259, Lys301, Glu323, Tyr343, and Tyr347, on inlA, and Val3, Pro5, Gln23, Lys25, and Asn27 on Ecad. In the inlA–hEcad complex, this cluster is also close to Ala281, Asn282, and Asn325 on inlA, and in the inlA–mEcad complex, it is close to Glu326 on inlA and Trp59 on Ecad. The cluster contains nine and eleven water sites in the inlA–hEcad and inlA–mEcad complex, respectively, with an average occupancy of the water sites of 770 and 669. This cluster will be denoted cluster 1.

A second cluster is close to residues Thr148, Phe150, Glu170, Ser192, Arg211, Asp213, Ser233, and Ile235, on inlA, and residues Pro/Glu16, and Phe17 Ecad. In addition, in the inlA–hEcad complex, this cluster is close to Arg168, Gln190, Ser233 and Glu255 on inlA, and Gly15, Lys19, and Asn20 on Ecad. In the inlA–mEcad complex, the cluster is close to Leu191 on inlA and Pro18 on Ecad. In the inlA–hEcad complex, this cluster contains twelve water sites that have an average occupancy of 838, and in the inlA–mEcad, the cluster contains eight water sites with an average occupancy of 686. This cluster will be referred to as cluster 2.

The smallest of the clusters, cluster 3, is close to residues Arg85, Asn107, and Asn128 on inlA, and residues Thr63 and Glu/Gln64 on Ecad. In addition, it is close to Asn104 and Ser106 on inlA in the inlA–hEcad complex. The cluster contains two water sites in the inlA–hEcad complex with an average occupancy of 958, and only one site in the inlA–mEcad complex, with an occupancy of 833. The water sites of cluster 1 and 3 are fairly consistent when comparing, inlA–hEcad and inlA–mEcad. However, the water sites in cluster 2 occupy partly different locations. In addition to the three clusters, there is a water site between Arg365 on inlA and Pro6 on Ecad, which is present in both the inlA–hEcad and inlA–mEcad complex, and one between Arg85 on inlA and Pro46, Pro47 and Val48 on Ecad, that is only present in the inlA–hEcad complex.

The residues close to the water sites form hydrogen bonds to water molecules found in most of the simulations, as shown in Table 5. For the inlA–hEcad complex, the average occupancy ranges from 22 to 200%, with an average of 76%, and for the inlA–mEcad complex the average is slightly lower at 57%. Most of the hydrogen bonds occur in both complexes, with the exception of hydrogen bonds to Arg85 on inlA in the inlA–mEcad complex, and hydrogen bonds to Glu16 on mEcad. The latter hydrogen bonds are naturally not possible in the inlA–hEcad complex.

**Table 5:** Hydrogen bonds between water molecules and residues close to conserved water sites.

		inlA–hEcad complex		inlA–mEcad complex	
Protein	Residue	Occurrence^a^	Occupancy^b^	Occurrence^a^	Occupancy^b^
**inlA**	Arg85^c^			9	19 ± 7.0
	Arg168	10	26.3 ± 2.2	10	27.4 ± 3.0
	Glu170	10	76.3 ± 8.7	10	30 ± 9.5
	Ser172	10	70.6 ± 6.4	10	60.1 ± 5.5
	Gln190	10	43.3 ± 7.1	10	30.4 ± 5.4
	Ser192	10	67.2 ± 8.2	10	89.8 ± 7.3
	Arg211	10	35.2 ± 7.7	10	23.5 ± 4.1
	Asp213	10	159.4 ± 25.1	10	124.9 ± 25.6
	Glu255	10	103.2 ± 16.1	10	105.7 ± 11.9
	Asn259	10	52.1 ± 6.3	10	62.3 ± 6.1
	Asn282	10	51.9 ± 5.2	10	47.2 ± 2.8
	Lys301	9	21.8 ± 7.8	10	20.4 ± 5.4
	Gln323	10	200.8 ± 15.1	10	204.3 ± 19.3
	Glu326	10	160 ± 10.4	10	108.1 ± 10.7
	Tyr343	8	55.6 ± 15.2	6	38.5 ± 13.0
	Tyr347	10	81.9 ± 3.5	10	72.3 ± 9.3
	Arg365	10	41.8 ± 4.3	10	30.9 ± 5.9

**Ecad**	Lys14	10	28.1 ± 3.9	10	29.5 ± 2.3
	Pro/Glu16			10	88.5 ± 15.9
	Lys19	10	38.5 ± 5.2	10	22.6 ± 4.1
	Gln23	10	37.1 ± 6.9	10	31.8 ± 6.7
	Lys25	10	43.5 ± 6.5	10	38.2 ± 4.2
	Asn27	10	73.3 ± 7.0	10	43.1 ± 10.7
	Thr63	10	51.5 ± 2.5	9	43.3 ± 10.1
	Glu/Gln64	10	138.3 ± 11.7	10	24.6 ± 4.0

a The number of simulations in which the hydrogen bond occurred

b The average number of snapshots the hydrogen bond was formed. This is averaged over all possible donor and acceptor atoms. Occupancy of more than 100% is possible because more than one water molecule can hydrogen bond to the same residue.

c This residue form a hydrogen bond to water molecules in inlA–hEcad as well but with a very low occupancy.

It is interesting to note that there is a “dry” region between cluster 1 and 2 (see Figure 6), where water molecules exchange readily with bulk water. This highlights that the interface between the two subunits is not contiguous.

### Extended simulations

To monitor the stability of the inlA–hEcad and inlA–mEcad complexes during a longer period of time, 200 ns simulations were performed for each of these. The simulations were performed in a slightly larger box allowing the proteins to diffuse in case of complex dissolution. The structural evolution of the complexes measured as the root mean square deviation after fitting each snapshot to the starting structure is shown in Table 6. To monitor the evolution, we made the fit based on the backbone atoms of inlA rather than the full complex. As such, the analysis will more easily reveal if the complex is separating or not. We will therefore only see a modest evolution of the inlA residues; the RMSD is in this case 1.4 to 1.6 Å for backbone atoms, and 1.7 to 1.9 Å for all heavy atoms. If we instead look at the Ecad atoms we observe larger deviations, and surprisingly, hEcad show larger deviations than mEcad, although inlA–hEcad should be a tighter complex. The RMS for hEcad is 2.7 Å for backbone atoms and 3.1 Å for all heavy atoms over the entire simulation. The corresponding measures for mEcad are 2.0 and 2.5 Å, respectively. However, looking at the two halves of the simulation individually, it is clear that most of the changes occur after 100 ns. Considering only the interfacial residues, it is clear that not all of the overall change comes from these residues, and that the RMSD in this region is similar between the two complexes.

**Table 6:** Structure evolution during the course of a 200 ns long simulation.

Residues	Atoms	Full simulation	First 100 ns	Last 100 ns
**inlA–hEcad complex**				
All on inlA	Backbone	1.6	1.5	1.6
	All heavy	1.9	1.8	1.9
All on Ecad	Backbone	2.7	2.1	3.3
	All heavy	3.1	2.6	3.6
Interfacial	Backbone	1.8	1.4	2.1
	All heavy	2.1	1.7	2.5

**inlA–mEcad complex**				
All on inlA	Backbone	1.4	1.4	1.4
	All heavy	1.7	1.7	1.8
All on Ecad	Backbone	2	1.8	2.2
	All heavy	2.5	2.2	2.7
Interfacial	Backbone	1.3	1.1	1.5
	All heavy	1.8	1.6	2

Measured as the root mean square deviation compared to the starting structure in Angstroms. The uncertainty of the measurements is below 0.01 Å.

We also calculated the MM/GBSA binding free energy of the complex for the last 10 ns of the simulations. The binding affinity for the inlA–hEcad complex is –183.1 kJ/mol, and –170.9 kJ/mol for the inlA–mEcad complex. The difference compared to the average over the 10 short simulations is significant for both complexes. This analysis shows that the structural evolution observed by the RMSD analysis leads to a looser inlA–hEcad complex and a tighter inlA–mEcad complex.

### Umbrella sampling

**Figure 7:**
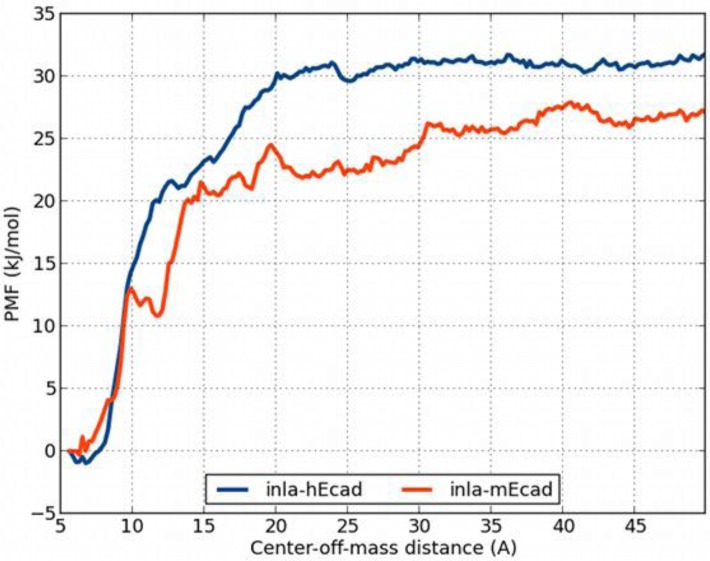
Average PMF for inlA–hEcad (blue) and inlA–mEcad (red).

To measure the binding strength between inlA and Ecad in an alternative way to MM/GBSA, we computed the potential of mean force (PMF) between the proteins using umbrella sampling and the weighted histogram analysis method. The direction in which Ecad was artificially displaced from inlA is illustrated in Figure 3. We also tried to displace Ecad in a perpendicular direction but the PMFs were very noisy (results not shown). The average PMFs for inlA–hEcad and inlA–mEcad are shown in Figure 7. We performed three independent sets of simulations for inlA–hEcad and two independent sets of simulations for inlA–mEcad. The PMFs are sufficiently converged at a center-of-mass distance of 50 Å, which implies that we could estimate a binding free energy from this point by taking the negative of the PMF (we set the PMF to zero at the displacement of 0 Å). Using this approach, the binding free energy of inlA–hEcad and inlA–mEcad is -32±6 and -27±1 kJ/mol, respectively. The estimates from the individual sets of simulations are given in Table 7. It is clear that the inlA–hEcad estimate is much more uncertain than the inlA–mEcad estimate, although the curve obtained for the inlA-mEcad system (Figure 7) is much more noise. Hence, the difference in binding affinity between the complexes, albeit indicating that inlA binds weaker to mEcad than to hEcad, should be taken with some caution.

**Table 7:** Binding strengths computed from potential of mean force in kJ/mol^a^.

	inlA–hEcad	inlA–mEcad
Set1	-21.8	-26.5
Set2	-30.4	-27.7
Set3	-43.2	
Average	-31.8±5.7	-27.1±0.6

a For inlA–hEcad, three independent sets of simulations was performed, and for inlA–mEcad two independent sets was performed

## Discussion

### The interface between inlA and hEcad

**Figure 8:**
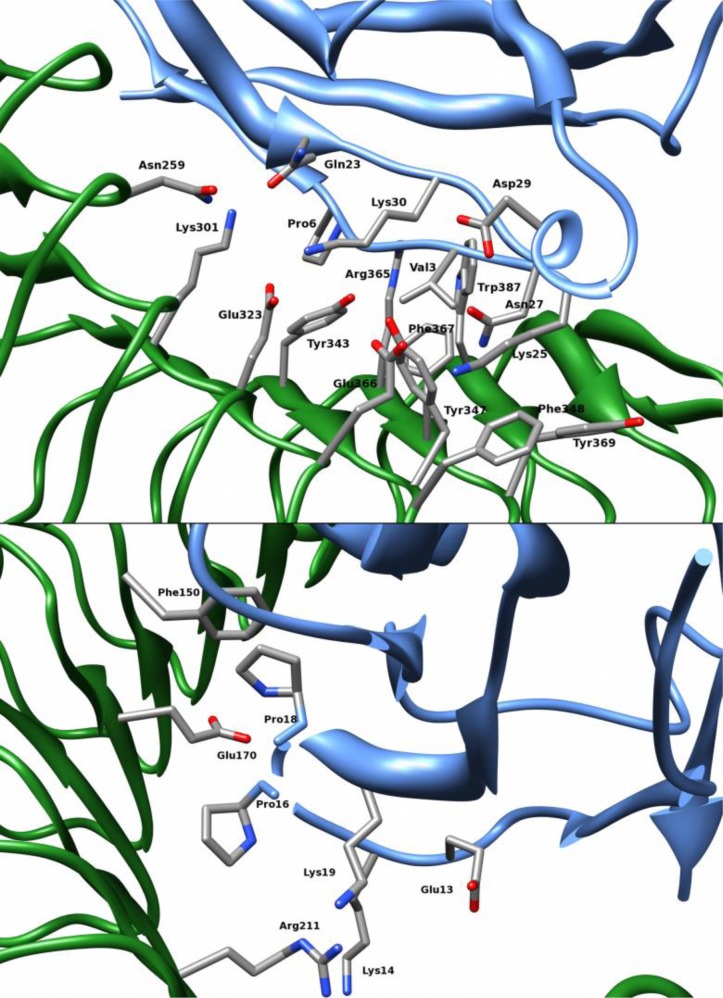
**Illustration of the two main clusters of hot spots**. inlA is shown in green and hEcad is shown in blue, hot spots are colored by atom.

The hot spots can be divided into two main clusters of residues. One of the clusters contains residues on LRR's 9, 11, 13, and 14 of inlA and residues on Ecad located on the loop close to the N-terminal, between β-sheets *b* and *c*, and on β-sheet *d* (see Figure 1 for numbering of β-sheets). These are the hot spots Asn259, Lys301, Glu323, Tyr343, Tyr347, Phe348, Arg365, Phe367, and Trp387 on inlA, and Val3, Pro6, Gln23, Lys25, Asp29, and Lys30 on Ecad. These residues are illustrated in Figure 8. Asn259, Lys301, and Glu323 on inlA and Lys25 and Trp59 on Ecad form a network of hydrogen bonds and charge–charge interactions. Of these, the residues on Ecad are most important for the binding. Furthermore, Tyr343 and Tyr347 are involved in stabilizing interfacial water sites and contribute a fair amount to the binding affinity. Lys30 forms a hydrogen bond with Glu326 on inlA, albeit not being a hot spot. This interaction is thus not important for the binding, although Lys30 does contribute.

The other main cluster of residues consists of residues on LRR's 4, 5 and 7 of inlA and residues on the loop between β-sheets *a* and *b* of Ecad, as illustrated in Figure 8. These are the hot spots Phe150, Glu170, and Arg211 on inlA and Glu13, Lys14, Pro16, Pro18, and Lys19 on Ecad. Arg211 on inlA forms a hydrogen bond with the backbone of Gly15 on Ecad and coordinates conserved water sites. Gly15 was shown to be important by ED, but cannot be analysed using ASM. Lys14 and Lys19 on Ecad also stabilize the conserved water sites. The important Pro16 residue on Ecad, contributing more than –30 kJ/mol to the binding free energy, forms unspecific, apolar contacts with the residues on inlA LRR 6 that forms a cavity-like structure. Pro16 and the surrounding residues are lined with conserved water sites. Pro18 on Ecad and Phe150 on inlA make apolar contacts.

Apart from these two clusters of residues and contacts, there are two additional hot spots. Arg85 of inlA forms a relatively well-conserved charge–charge interaction with Glu64 on Ecad, which is not a hot spot. Arg85 also stabilize two water sites and contributes with –39 kJ/mol to the binding affinity. Lastly, Glu56 does not have any clear binding partner and it is unclear why this residue should be important for the binding.

The hot spots on inlA contribute –118 kJ/mol binding affinity, and those on Ecad contribute –104 kJ/mol. Divided into the clusters of residues discussed above, the residues on inlA in cluster 1 contribute –71 kJ/mol and those on Ecad contribute –35 kJ/mol. The residues on the second cluster contribute –16 and –50 kJ/mol, for the inlA and Ecad residues, respectively. This indicates that most of the binding affinity comes from these two clusters, although there are a few other separate residues that also contribute greatly thereto (such as Arg85). It is also interesting that inlA contributes mostly through the residues in the first cluster, but Ecad contributes mostly through the residues in the second cluster.

### The interface between inlA and mEcad

The hot spots of the interface in this system are to a large degree equivalent to those in the inlA–hEcad complex. This is interesting to note as the interface between inlA and wild-type mEcad has been only partially characterized by experiments, due to the inability to crystalize the complex. Hence, this study complements existing literature.

The cluster of residues close to the LRR β-sheets 9, 11, 13, and 14 of inlA also includes Glu326 (which forms a hydrogen bond with Lys25) as well as Tyr369 (which forms a non-specific interaction with Asn27 on Ecad). At least the sASM analysis suggests that Asn27 should be considered as a hot spot. This interaction has been discussed much in the literature, and it is argued that it is favorable to mutate Tyr369 to serine. However, the simulations with the Y369S mutant did not result in any improved binding affinity. The hot spot residues on the loop close to the N-terminal, residues on and between β-sheets *b* and *c*, and residues on β-sheet *d* of mEcad are identical to the residues in inlA–hEcad.

The second cluster, located on the LRR β-sheets 4, 5 and 7 of inlA and on the loop between β-sheets *a* and *b* of Ecad, differs more. The largest difference comes from the substitution of Pro16 to Glu16. It contributes as much as 17 kJ/mol less to the binding affinity than Pro16 in the inlA–hEcad complex, but the contribution is nonetheless favorable. However, instead of protruding into the apolar cavity of LRR 6 it bends outwards and forms stable hydrogen bonds with Arg211. Instead, the cavity seems to be filled with conserved water sites. Pro18 on Ecad and Phe150 on inlA make apolar contacts, similar to those in the inlA–hEcad complex. This is has not been described experimentally, and show that although the cavity is unfavorabe for Glu16 (as hypothesis by experiment and confirmed here), the protein is able to adapt and form new interactions. Two hot spots, Arg168 on inlA and Glu13/31 on Ecad make non-specific contacts and do not interact directly with the opposite protein.

Lastly, Arg85 on inlA forms consistent hydrogen bonds with Gln64 on Ecad, and in the inlA–mEcad complex both residues are hot spots.

The hot spots on inlA and Ecad contribute –137 and –50 kJ/mol, respectively. Looking at residues in the first cluster only, the contributions are –67 and–29 kJ/mol, for inlA and Ecad, respectively, whereas in the second cluster the residues on inlA contribute –51 kJ/mol, and the residues on mEcad –20 kJ/mol. mEcad thus provides a much weaker interaction (30 kJ/mol less) in the second cluster, than what hEcad does. In the inlA-hEcad complex, hEcad is the dominating contributor of this cluster.

## Conclusions

We have performed simulations of *L. monocytogenes* internalin A (inlA) and either human or murine E-cadherin (Ecad). Both the wild type and various mutants have been simulated. Although the different methods to analyze the interfacial residues give somewhat ambiguous results, we believe that a lot of useful information is provided with regards to the energetics of the interaction. The interfaces of the two complexes are very similar and there are small differences that result in the apparent lower binding affinity for the inlA–mEcad complex. The two proteins bind together using two large clusters of residues, in addition to one smaller cluster. One of the two large clusters is more or less identical in the two complexes, and all the difference in binding affinity stems from the other two clusters. The substitution of Pro16 on hEcad to Glu16 on mEcad, shifts the hydrogen-bonding partners and conserved water sites. While Pro16 in hEcad protrudes into an apolar cavity at LRR 6 of inlA that is lined with conserved water sites, Glu16 in mEcad bends outside the cavity to form hydrogen bonds with Arg211 on inlA, thereby pushing the water molecules towards the cavity. It is clear that the latter configuration of water sites is less favorable than the former, as shown by the much lower occupancy. The mutant simulations clearly show that the binding affinity is lowered when Pro16 is mutated to Glu, and that a Glu16 to Pro mutation strengthens the affinity.

The last cluster of important residues is mainly formed by interactions between Arg85 on inlA and Glu/Gln64 on Ecad, and a number of conserved water sites. In the inlA–hEcad complex, Arg85 and Glu64 is able to form a tight salt bridge that is also able to attract more water molecules, whereas in inlA–mEcad, there is a single hydrogen bond between Arg85 and Gln64 and fewer water molecules. That the salt bridge interaction is favorable was clearly shown in the mutant simulations, where a E64Q mutation in inlA–hEcad considerably lowered the binding affinity, whereas the Q64E mutation in inlA–mEcad strengthened the binding affinity. However, due to weaker binding of the cluster containing Glu16 *vs*. that containing Pro16, the interaction between Arg85 and Gln64 is of higher relative importance in inlA–mEcad than in the inlA–hEcad E64Q mutant.

Up to this point, we have confirmed the experimental observation that inlA–mEcad is a weaker complex than inlA–hEcad. This observation has been used as the main argument to explain why the bacterium is unable to invade murine cells, while it can invade human cell. However, we have performed our simulations from crystal structures of the already formed complex. To this end we performed 200 ns simulations of the inlA–hEcad and inlA–mEcad complexes and showed that the observed differences are not sufficient for the dissolution of the inlA–mEcad complex. Contrary, we observe larger changes for the inlA–hEcad complex. The umbrella sampling simulations also corroborate this observation. The binding strength estimated from these simulations show no significant difference between inlA–hEcad and inlA–mEcad. However, it must be noted that we have judiciously chosen one dissociation pathway, and that more than one pathway may exist. The umbrella sampling is fundamentally different to MM/GBSA so it should not come as a surprise that they indicate different relative free energies. Still, based on the results in this study we cannot attribute the inability of *L. monocytogenes* to invade murine to the interactions between the inlA and mEcad at the nanosecond to sub-microsecond timescale (the time scales of our simulations). Either, the processes involved occur on a much longer timescale than is readily accessible with conventional simulations or there is some hitherto unknown mechanism that precludes the binding from taking place altogether. One possible reason could be that the unbound structures of mEcad and hEcad differ substantially such that mEcad cannot be properly presented for inlA to bind. Our conclusion is interesting as it questions an important hypothesis regarding *L. monocytogenes* invasion.

## Supplementary Material

Of mice and men: Dissecting the interaction between *Listeria monocytogenes* Internalin A and E-cadherin

## References

[1] Bierne H , Cossart P (2007) Listeria monocytogenes surface proteins: from genome predictions to function. Microbiol mol biol rev 71: 377–397 17554049 10.1128/MMBR.00039-06 PMC1899877

[2] Vazquez-Boland JA , Kuhn M , Berche P , Chakraborty T , Dominguez-Bernal G , et al. (2001) Listeria Pathogenesis and Molecular Virulence Determinants. Clinic Microbiol Rev 14: 584–640 10.1128/CMR.14.3.584-640.2001 PMC88991 11432815

[3] Bonazzi M , Lecuit M , Cossart P (2009) Listeria monocytogenes internalin and E-cadherin: from bench to bedside. Cold Spring Harb Perspect Biol 1: a003087 20066101 10.1101/cshperspect.a003087 PMC2773623

[4] Hamon M , Bierne H , Cossart P (2006) Listeria monocytogenes: a multifaceted model. Nat rev Microbiol 4: 423–434 16710323 10.1038/nrmicro1413

[5] Bierne H , Sabet C , Personnic N , Cossart P (2007) Internalins: a complex family of leucine-rich repeat-containing proteins in Listeria monocytogenes. Microbes Infect 9: 1156–1166 17764999 10.1016/j.micinf.2007.05.003

[6] Schubert WD , Urbanke C , Ziehm T , Beier V , Machner MP , et al. (2002) Structure of internalin, a major invasion protein of Listeria monocytogenes, in complex with its human receptor E-cadherin. Cell 111: 825–836 12526809 10.1016/s0092-8674(02)01136-4

[7] Disson O , Nikitas G , Grayo S , Dussurget O , Cossart P , et al. (2009) Modeling human listeriosis in natural and genetically engineered animals. Nat Prot 4: 799–810 10.1038/nprot.2009.66 19444238

[8] Hoelzer K , Pouillot R , Dennis S (2012) Animal models of listeriosis: a comparative review of the current state of the art and lessons learned. Vet Res 43: 18 22417207 10.1186/1297-9716-43-18 PMC3384455

[9] Wollert T , Heinz DW , Schubert W-D (2007) Thermodynamically reengineering the listerial invasion complex InlA/E-cadherin. Proc Nat Ac Sc USA 104: 13960–13965 10.1073/pnas.0702199104 PMC1955803 17715295

[10] Wollert T , Pasche B , Rochon M , Deppenmeier S , van den Heuvel J , et al. (2007) Extending the host range of Listeria monocytogenes by rational protein design. Cell 129: 891–902 17540170 10.1016/j.cell.2007.03.049

[11] Massova I , Kollman PA (1999) Computational Alanine Scanning To Probe Protein-Protein Interactions: A Novel Approach To Evaluate Binding Free Energies. J Am Chem Soc 121: 8133–8143

[12] Zoete V , Michielin O (2007) Comparison Between Computational Alanine Scanning and Per-Residue Binding Free Energy Decomposition for Protein – Protein Association Using MM-GBSA: Application to the TCR-p-MHC Complex. Proteins 67: 1026–1047 17377991 10.1002/prot.21395

[13] Moreira IS , Fernandes PA , Ramos MJ (2007) Hot spots—A review of the protein–protein interface determinant amino-acid residues. Proteins 68: 803–812 17546660 10.1002/prot.21396

[14] Metz A , Pfleger C , Kopitz H , Pfeiffer-Marek S , Baringhaus K-H , et al. (2012) Hot spots and transient pockets: predicting the determinants of small-molecule binding to a protein-protein interface. J Chem Inf Model 52: 120–133 22087639 10.1021/ci200322s

[15] Lindorff-Larsen K , Piana S , Palmo K , Maragakis P , Klepeis JL , et al. (2010) Improved side-chain torsion potentials for the Amber ff99SB protein force field. Proteins 78: 1950–1958 20408171 10.1002/prot.22711 PMC2970904

[16] Jorgensen WL , Chandrasekhar J , Madura JD , Impey RW , Klein ML (1983) Comparison of simple potential functions for simulating liquid water. J Chem Phys 79: 926

[17] Hess B , Kutzner C , van der Spoel D , Lindahl E (2008) GROMACS 4: Algorithms for Highly Efficient, Load-Balanced, and Scalable Molecular Simulation. J Chem Theory Comput 4: 435–447 10.1021/ct700301q 26620784

[18] Hess B , Bekker H , Berendsen HJC , Fraaije JGEM (1997) LINCS: A linear constraint solver for molecular simulations. J Comput Chem 18: 1463–1472

[19] Darden T , York D , Pedersen L (1993) Particle mesh Ewald: An Nlog(N) method for Ewald sums in large systems. J Chem Phys 98: 10089

[20] Allen MP , Tildesley DJ (1987) Computer Simulations of Liquids. Oxford: Oxford Science Publications

[21] Bussi G , Donadio D , Parrinello M (2007) Canonical sampling through velocity rescaling. J Chem Phys 126: 014101 17212484 10.1063/1.2408420

[22] Berendsen HJC , Postma JPM , van Gunsteren WF , DiNola A , Haak JR (1984) Molecular dynamics with coupling to an external bath. J Chem Phys 81: 3684

[23] Genheden S , Ryde U (2011) A comparison of different initialization protocols to obtain statistically independent molecular dynamics simulations. J Comput Chem 32: 187–195 21132839 10.1002/jcc.21546

[24] Srinivasan J , Cheatham TE , Cieplak P , Kollman PA , Case DA (1998) Continuum Solvent Studies of the Stability of DNA, RNA, and Phosphoramidate-DNA Helices. J Am Chem Soc 120: 9401–9409

[25] Miller BR , McGee TD , Swails JM , Homeyer N , Gohlke H , et al. (2012) MMPBSA.py: An Efficient Program for End-State Free Energy Calculations. J Chem Theory Comput 8: 3314–3321 10.1021/ct300418h 26605738

[26] Genheden S , Ryde U (2012) Comparison of end-point continuum-solvation methods for the calculation of protein-ligand binding free energies. Proteins 80: 1326–1342 22274991 10.1002/prot.24029

[27] Moreira IS , Fernandes PA , Ramos MJ (2006) Unravelling Hot Spots: a comprehensive computational mutagenesis study. Theor Chem Acc 117: 99–113

[28] Onufriev A , Bashford D , Case DA (2004) Exploring protein native states and large-scale conformational changes with a modified generalized born model. Proteins 55: 383–394 15048829 10.1002/prot.20033

[29] Kuhn B , Kollman PA (2000) Binding of a Diverse Set of Ligands to Avidin and Streptavidin: An Accurate Quantitative Prediction of Their Relative Affinities by a Combination of Molecular Mechanics and Continuum Solvent Models. J Med Chem 43: 3786–3791 11020294 10.1021/jm000241h

[30] Moreira IS , Fernandes PA , Ramos MJ (2006) Unraveling the importance of protein-protein interaction: application of a computational alanine-scanning mutagenesis to the study of the IgG1 streptococcal protein G (C2 fragment) complex. J Phys Chem B 110: 10962–10969 16771349 10.1021/jp054760d

[31] Young T , Abel R , Kim B , Berne BJ , Friesner RA (2007) Motifs for molecular recognition exploiting hydrophobic enclosure in protein-ligand binding. Proc Nat Ac Sc USA 104: 808–813 10.1073/pnas.0610202104 PMC1783395 17204562

[32] Lazaridis T (1998) Inhomogeneous Fluid Approach to Solvation Thermodynamics. 1. Theory. J Phys Chem B 102: 3531–3541

[33] Li Z , Lazaridis T (2003) Thermodynamic contributions of the ordered water molecule in HIV-1 protease. J Am Chem Soc 125: 6636–6637 12769565 10.1021/ja0299203

[34] Nguyen CN , Young TK , Gilson MK (2012) Grid inhomogeneous solvation theory: hydration structure and thermodynamics of the miniature receptor cucurbit[7]uril. J Chem Phys 137: 044101 22852591 10.1063/1.4733951 PMC3416872

[35] Torrie GM , Valleau JP (1977) Nonphysical sampling distributions in Monte Carlo free-energy estimation: Umbrella sampling. J Comput Phys 23: 187–199

[36] Kumar S , Rosenberg JM , Bouzida D , Swendsen RH , Kollman PA (1992) The weighted histogram analysis method for free-energy calculations on biomolecules. I. The method. J Comput Chem 13: 1011–1021

[37] Hub JS , de Groot BL , van der Spoel D (2010) g_wham—A Free Weighted Histogram Analysis Implementation Including Robust Error and Autocorrelation Estimates. J Chem Theory Comput 6: 3713–3720

