# Supplementary material for: Of mice and men: Dissecting the interaction between Listeria monocytogenes Internalin A and E-cadherin
Source: Comput Struct Biotechnol J. 2013 Dec 15;6:e201303022. doi: 10.5936/csbj.201303022 (PMC3962206; doi:10.5936/csbj.201303022)
Supplement: Of mice and men: Dissecting the interaction between Listeria monocytogenes Internalin A and E-cadherin [file CSBJ-6-e201303022_SM0001.pdf]

Supporting Information:

Of mice and men: Dissecting the interaction between *Listeria monocytogenes* Internalin A and E-cadherin

Samuel Genheden[a], Leif A. Eriksson

Division of Physical Chemistry, Department of Chemistry and Molecular Biology, University of Gothenburg
Kemivägen 10, SE-412 96 Göteborg, Sweden

[a] Present address: School of Chemistry, University of Southampton, Highfield, SO17 1BJ, Southampton, UK.

Correspondence to Samuel Genheden, E-mail: s.genheden@soton.ac.uk

Table S1. RMSD analysis of MM/GBSA simulations[a]

Simulation	All on inlA		All on Ecad		Interfacial	
	Backbone	All heavy	Backbone	All heavy	Backbone	All heavy
			inlA–hEcad simulations			
1	1.1	1.3	1.4	1.7	0.8	1.0
2	1.3	1.5	1.4	1.7	1.1	1.3
3	1.0	1.2	1.3	1.6	0.9	1.2
4	0.9	1.2	1.5	1.8	1.1	1.2
5	1.0	1.2	1.1	1.4	0.8	1.0
6	1.1	1.3	1.1	1.4	0.9	1.1
7	0.9	1.1	1.0	1.3	0.7	0.9
8	0.9	1.2	1.3	1.5	0.9	1.1
9	1.1	1.3	1.4	1.6	1.1	1.3
10	1.0	1.2	1.2	1.4	0.8	1.0
			inlA-mEcad simulations			
1	1.2	1.4	2.0	2.2	1.2	1.4
2	1.3	1.5	1.5	1.8	1.2	1.4
3	1.3	1.5	1.3	1.6	0.9	1.1
4	1.1	1.3	1.9	2.1	1.1	1.3
5	1.1	1.3	1.4	1.6	1.0	1.2
6	1.1	1.4	1.5	1.7	1.0	1.2
7	1.1	1.3	1.3	1.6	0.8	1.0
8	1.0	1.2	1.1	1.4	0.8	1.1
9	1.0	1.2	1.2	1.5	0.8	1.0
10	1.5	1.7	1.6	1.8	1.3	1.4

[a]The root mean square deviation compared to the starting structure in Angstroms. The uncertainty of the measurements is below 0.02 Å.

Table S2. Free energy contributions of residues on inlA in kJ/mol[a]

	inlA–hEcad complex			inlA–mEcad complex			Difference[b]		
	ED	ASM	sASM	ED	ASM	sASM	ED	ASM	sASM
Asp84	5.7 ±0.0	4.7 ±0.1	3.5 ±0.1	4.8 ±0.1	3.2 ±0.3	2.2 ±0.1	0.9	1.4	1.3
Arg85	-38.9 ±0.6	-72.6 ±1.1	-39.7 ±0.3	-20.8 ±0.3	-52.8 ±0.7	-34.9 ±0.4	-18.1	-19.8	-4.8
Phe150	-15.3 ±0.1	-24.8 ±0.2	-25.8 ±0.2	-15.5 ±0.2	-25.5 ±0.3	-27.0 ±0.3	0.2	0.7	1.2
Arg168	-2.9 ±0.1	-8.2 ±0.2	-6.9 ±0.2	-5.7 ±0.2	-12.2 ±0.3	-12.6 ±0.4	2.7	4.0	5.7
Glu170	6.6 ±0.2	-13.6 ±0.2	-11.8 ±0.2	5.1 ±0.3	-13.9 ±0.6	-9.7 ±0.1	1.5	0.3	-2.1
Ser172	0.9 ±0.2	1.7 ±0.1	-0.8 ±0.1	1.0 ±0.0	1.2 ±0.1	-1.2 ±0.0	-0.1	0.5	0.4
Gln190	-2.1 ±0.2	-6.7 ±0.3	-15.6 ±0.1	-6.6 ±1.3	-9.8 ±2.0	-9.7 ±0.3	4.6	3.1	-5.9
Leu191	-2.4 ±0.1	-0.8 ±0.0	-0.6 ±0.0	-1.0 ±0.1	-0.3 ±0.0	-0.2 ±0.0	-1.4	-0.5	-0.4
Ser192	-1.9 ±0.2	0.2 ±0.5	-1.9 ±0.2	-0.6 ±0.1	-0.6 ±0.1	-0.9 ±0.0	-1.3	0.8	-1.0
Arg211	-7.6 ±0.6	-14.2 ±1.8	-19.9 ±0.8	-23.4 ±1.8	-32.2 ±2.7	-14.6 ±0.3	15.8	18.0	-5.3
Asp213	4.7 ±0.2	4.6 ±0.2	-2.3 ±0.1	3.8 ±0.1	2.1 ±0.3	-0.2 ±0.2	0.9	2.5	-2.1
Ile235	-4.3 ±0.2	-3.5 ±0.1	-4.6 ±0.2	-1.9 ±0.2	-1.7 ±0.2	-2.3 ±0.2	-2.3	-1.7	-2.4
Glu255	-1.8 ±1.3	-10.3 ±3.0	-3.4 ±0.5	4.2 ±0.2	3.9 ±0.3	1.8 ±0.1	-6.0	-14.1	-5.2
Asn259	-7.1 ±0.5	-16.8 ±1.2	-11.6 ±0.5	-4.0 ±0.7	-9.7 ±1.6	-8.5 ±0.8	-3.1	-7.0	-3.1
Asp277	4.6 ±0.1	5.3 ±0.2	2.0 ±0.1	4.1 ±0.1	4.9 ±0.1	2.2 ±0.1	0.5	0.4	-0.2
Asp279	0.8 ±0.7	-12.7 ±1.6	-4.7 ±0.3	0.7 ±0.2	-11.1 ±0.6	-4.1 ±0.1	0.1	-1.6	-0.6
Asn282	0.6 ±0.4	-0.1 ±1.1	-1.7 ±0.5	1.1 ±0.0	2.3 ±0.1	-0.1 ±0.2	-0.5	-2.4	-1.6
Glu299	6.5 ±0.2	7.1 ±0.3	2.5 ±0.1	5.9 ±0.3	6.5 ±0.3	2.4 ±0.1	0.7	0.6	0.1
Lys301	7.2 ±0.9	-15.2 ±1.7	-11.3 ±0.7	3.7 ±0.8	-15.6 ±1.4	-10.7 ±0.4	3.5	0.4	-0.6
Glu323	-1.5 ±1.7	-20.1 ±4.3	-7.6 ±1.0	7.4 ±0.2	4.5 ±0.3	-1.7 ±0.2	-8.9	-24.6	-6.0
Glu326	1.7 ±0.6	-8.5 ±1.5	-5.5 ±0.3	-4.5 ±1.3	-25.8 ±3.4	-10.1 ±0.8	6.3	17.3	4.6
Tyr343	-13.2 ±1.1	-27.9 ±1.7	-26.3 ±0.9	-12.3 ±1.2	-25.4 ±1.6	-24.9 ±0.4	-0.8	-2.5	-1.4
Tyr347	-9.9 ±0.9	-16.5 ±1.1	-18.6 ±0.8	-8.9 ±0.8	-15.8 ±1.3	-17.3 ±0.7	-0.9	-0.7	-1.3
Phe348	-9.0 ±0.3	-12.6 ±0.4	-14.0 ±0.4	-9.4 ±0.4	-13.3 ±0.5	-14.5 ±0.6	0.4	0.7	0.5
Arg365	-10.7 ±0.6	-29.4 ±0.6	-30.1 ±0.4	-9.3 ±1.1	-26.5 ±2.1	-27.3 ±1.2	-1.4	-3.0	-2.8
Phe367	-11.2 ±0.3	-19.0 ±0.4	-19.5 ±0.4	-10.9 ±0.7	-18.7 ±1.1	-19.2 ±1.1	-0.3	-0.3	-0.3
Tyr369	-6.2 ±1.0	-6.2 ±1.3	-10.7 ±1.0	-7.4 ±0.6	-8.5 ±0.7	-10.9 ±0.5	1.2	2.2	0.3
Trp387	-9.3 ±0.4	-19.4 ±0.8	-22.7 ±1.0	-8.7 ±0.7	-18.0 ±1.5	-21.2 ±1.6	-0.6	-1.4	-1.5
Gln409	1.0 ±0.1	-0.5 ±0.2	-5.0 ±0.2	1.1 ±0.2	0.0 ±0.3	-4.1 ±0.5	-0.1	-0.5	-0.9

[a] Residues where selected based on a number of criteria as outlined in the text. Free energy contributions are determined by energy decomposition (ED), alanine scanning mutagenesis (ASM), and scaled ASM (sASM).

[b] A positive difference indicates that the residue has a more positive free energy in inlA–hEcad than in inlA–mEcad.

Table S3. Free energy contributions of residues on Ecad in kJ/mol[a]

	inlA–hEcad complex			inlA–mEcad complex			Difference[b]		
	ED	ASM	sASM	ED	ASM	sASM	ED	ASM	sASM
Ser1	10.3 ±0.5			8.7 ±0.6			1.6		
Trp2	0.1 ±0.1	-1.4 ±0.1	-1.6 ±0.1	0.3 ±0.1	-1.1 ±0.1	-1.4 ±0.1	-0.2	-0.2	-0.2
Val3	-17.7 ±0.8	-12.5 ±0.3	-13.7 ±0.4	-17.4 ±1.0	-11.0 ±0.3	-12.5 ±0.4	-0.3	-1.5	-1.1
Ile4	-13.0 ±0.5	-4.2 ±0.2	-5.2 ±0.2	-13.7 ±0.9	-4.3 ±0.2	-5.4 ±0.3	0.7	0.1	0.2
Pro5	-12.0 ±0.3	-6.5 ±0.3	-6.8 ±0.3	-10.9 ±0.5	-6.1 ±0.3	-6.5 ±0.3	-1.1	-0.4	-0.2
Pro6	-12.9 ±0.6	-11.1 ±0.7	-11.8 ±0.7	-11.9 ±0.6	-10.3 ±0.6	-11.0 ±0.7	-1.0	-0.7	-0.8
Glu11	4.6 ±0.0	8.0 ±0.0	7.6 ±0.0	4.3 ±0.0	7.5 ±0.0	7.1 ±0.0	0.4	0.5	0.5
Glu13	10.4 ±0.2	14.7 ±0.3	11.4 ±0.1	8.9 ±0.3	13.3 ±0.3	10.5 ±0.1	1.5	1.4	0.9
Lys14	-12.7 ±2.0	-18.0 ±3.8	-23.0 ±1.6	-2.2 ±0.3	-7.2 ±0.2	-10.4 ±0.4	-10.5	-10.8	-12.6
Gly15	-7.1 ±0.4			0.7 ±0.5			-7.8		
Pro/Glu16	-32.6 ±0.2	-19.8 ±0.2	-22.3 ±0.1	-15.5 ±1.8	-35.4 ±5.3	-10.6 ±0.8	-17.1	15.6	-11.6
Phe17	-14.6 ±0.2	-3.4 ±0.1	-4.9 ±0.1	-10.2 ±0.2	-3.1 ±0.1	-3.8 ±0.1	-4.4	-0.3	-1.1
Pro18	-6.8 ±0.1	-9.5 ±0.3	-10.4 ±0.3	-7.6 ±0.1	-9.6 ±0.1	-10.2 ±0.1	0.8	0.1	-0.2
Lys19	-1.7 ±0.6	-10.4 ±1.5	-18.9 ±0.5	1.4 ±0.9	-8.2 ±1.0	-13.6 ±0.5	-3.1	-2.2	-5.3
Gln23	-11.7 ±1.7	-22.9 ±2.9	-13.7 ±1.4	-8.0 ±1.0	-17.3 ±2.0	-10.7 ±0.8	-3.7	-5.7	-3.0
Lys25	-3.8 ±2.5	-17.8 ±4.6	-22.8 ±1.2	-3.7 ±1.2	-15.0 ±2.8	-19.1 ±0.7	-0.1	-2.7	-3.7
Asn27	0.0 ±1.1	-5.0 ±2.3	-12.3 ±1.7	0.4 ±0.4	-5.1 ±0.7	-11.1 ±0.8	-0.5	0.1	-1.2
Asp29	9.9 ±0.5	13.5 ±1.1	9.1 ±0.4	11.6 ±0.3	17.6 ±0.6	10.3 ±0.1	-1.8	-4.1	-1.2
Lys30	-16.9 ±1.1	-29.7 ±2.5	-26.1 ±1.1	-16.7 ±1.0	-29.0 ±1.3	-27.1 ±0.6	-0.2	-0.8	1.1
Glu31	7.0 ±0.3	10.9 ±0.2	7.8 ±0.1	7.0 ±0.2	10.9 ±0.2	8.0 ±0.1	-0.1	0.1	-0.2
Asp44	3.9 ±0.1	6.7 ±0.1	6.4 ±0.0	3.7 ±0.1	6.3 ±0.2	6.2 ±0.1	0.2	0.4	0.2
Val48	-18.7 ±0.7	-5.1 ±0.4	-5.9 ±0.3	-19.4 ±0.6	-4.9 ±0.3	-5.6 ±0.3	0.7	-0.2	-0.2
Val50	-5.3 ±0.1	-2.3 ±0.1	-2.3 ±0.0	-5.5 ±0.2	-2.0 ±0.2	-2.3 ±0.1	0.1	-0.3	-0.1
Glu54	12.9 ±0.7	7.8 ±2.3	5.8 ±0.5	12.4 ±0.7	15.9 ±0.9	7.1 ±0.3	0.5	-8.1	-1.3
Glu56	8.0 ±0.1	11.5 ±0.1	8.4 ±0.1	8.4 ±0.2	11.5 ±0.2	8.2 ±0.2	-0.4	0.0	0.1
Trp59	-15.5 ±0.5	-21.4 ±0.9	-24.6 ±0.7	-12.3 ±0.9	-17.4 ±1.6	-22.5 ±1.5	-3.1	-4.0	-2.1
Thr63	-0.7 ±0.1	-4.0 ±0.1	-4.7 ±0.1	-1.6 ±0.1	-3.9 ±0.3	-4.5 ±0.3	0.9	-0.1	-0.2
Glu/Gln64	0.3 ±0.3	-23.5 ±0.9	-2.1 ±0.2	-7.0 ±0.3	-15.0 ±0.6	-8.2 ±0.2	7.4	-8.6	6.1
Asp67	4.5 ±0.1	8.1 ±0.1	7.3 ±0.1	4.0 ±0.0	7.4 ±0.1	6.8 ±0.0	0.5	0.7	0.5
Asp90	5.2 ±0.2	8.1 ±0.2	6.3 ±0.1	4.9 ±0.1	7.8 ±0.1	6.3 ±0.1	0.3	0.4	0.1
Met92	-5.3 ±0.2	-6.0 ±0.2	-8.7 ±0.3	-4.1 ±0.6	-4.8 ±0.7	-6.9 ±0.9	-1.1	-1.2	-1.8
Glu93	4.6 ±0.1	6.8 ±0.1	5.1 ±0.0	4.5 ±0.1	6.7 ±0.1	5.1 ±0.1	0.2	0.1	0.0
Asp100	4.1 ±0.1	7.6 ±0.1	7.2 ±0.1	3.6 ±0.0	6.9 ±0.1	6.6 ±0.1	0.5	0.8	0.6
Gln101	5.3 ±0.2			4.0 ±0.1			1.4		

[a] Residues where selected based on a number of criteria as outlined in the text. Free energy contributions are determined by energy decomposition (ED), alanine scanning mutagenesis (ASM), and scaled ASM (sASM).

[b] A positive difference indicates that the residue has a more positive free energy in inlA–hEcad than in inlA–mEcad.
